# Cryptochromes and the Circadian Clock: The Story of a Very Complex Relationship in a Spinning World

**DOI:** 10.3390/genes12050672

**Published:** 2021-04-29

**Authors:** Loredana Lopez, Carlo Fasano, Giorgio Perrella, Paolo Facella

**Affiliations:** Italian National Agency for New Technologies, Energy and Sustainable Economic Development (ENEA), TERIN-BBC-BBE, Trisaia Research Center, 75026 Rotondella, Matera, Italy; loredana.lopez@enea.it (L.L.); carlo.fasano@enea.it (C.F.); giorgio.perrella@enea.it (G.P.)

**Keywords:** circadian clock, light, cryptochromes, chromatin, epigenetic, cell cycle, gene expression regulation, *Arabidopsis*

## Abstract

Cryptochromes are flavin-containing blue light photoreceptors, present in most kingdoms, including archaea, bacteria, plants, animals and fungi. They are structurally similar to photolyases, a class of flavoproteins involved in light-dependent repair of UV-damaged DNA. Cryptochromes were first discovered in *Arabidopsis thaliana* in which they control many light-regulated physiological processes like seed germination, de-etiolation, photoperiodic control of the flowering time, cotyledon opening and expansion, anthocyanin accumulation, chloroplast development and root growth. They also regulate the entrainment of plant circadian clock to the phase of light–dark daily cycles. Here, we review the molecular mechanisms by which plant cryptochromes control the synchronisation of the clock with the environmental light. Furthermore, we summarise the circadian clock-mediated changes in cell cycle regulation and chromatin organisation and, finally, we discuss a putative role for plant cryptochromes in the epigenetic regulation of genes.

## 1. Introduction

Light is a vital environmental signal for plant development and physiology that activates an array of photoreceptors, each getting activated in response to a particular wavelength, followed by interaction between various signalling pathways, finally culminating in affecting gene expression. Plants possess a multiplicity of photoreceptors that allow them to recognize changes in the direction, intensity, and quality of light, including UV RESISTANCE LOCUS 8 (UVR8), phototropins (PHOTs), cryptochromes (CRYs), FLAVIN-BINDING KELCH REPEAT F-BOX 1 (FKF1), ZEITLUPE (ZTL), LOV KELCH PROTEIN 2 (LKP2) and phytochromes (PHYs) [1,2,3,4,5]. Among these, CRYs are the only photoreceptors present in all evolutionary lineages ranging from bacteria to mammals [6]. They are flavoproteins, structurally similar to DNA photolyases. Photolyases repair ultraviolet—induced DNA damage by a mechanism known as photoreactivation, using photons absorbed from the blue end of the light spectrum. CRYs can regulate plant light morphogenesis, flowering time and circadian clock in all major evolutionary kingdom. Furthermore, recent reports have highlighted their roles in controlling other aspects of plant development such as seed dormancy and germination, stomatal opening and development, photosynthetic reactions, stress responses and many others [7].

The majority of the pioneering studies on plant CRYs have been conducted in *A. thaliana*, which contains one member of each CRY subfamily: *CRY1*, *CRY2* and *CRY-DASH*, so called because it was found in *Drosophila*, *Arabidopsis*, *Synechocystis* and *Homo* [8,9,10]. In the subsequent years, CRYs have been identified in other plants, both dicots and monocots, and in some lower plant species such as algae and ferns. Two *CRY1* (*CRY1a* and *CRY1b*) members, one *CRY2* and one *CRY-DASH* member of each subfamily were reported in tomato [11,12]. In this dicot, functional characterisation studies in CRY2 overexpressing plants showed shortened hypocotyl and internodes under blue light like in *Arabidopsis*. In addition, CRY2 overexpressing tomato plants exhibited overproduction of anthocyanin and chlorophyll in leaves, flavonoids and lycopene in fruits [13]. Interestingly, the tomato *CRY-DASH* gene was found to be under circadian control [12]. In rice, the *CRY* gene family comprises two *CRY1* (*CRY1a* and *CRY1b*) genes and one *CRY2* gene [14]. Functional characterisation of rice CRYs revealed an increased blue light responsiveness of transgenics overexpressing CRY1, with coleoptile growth inhibition and repression of growth of leaf sheath and leaf blade, and the role of CRY2 in promoting flowering time [15]. The *CRY* gene family in wheat comprises two *CRY1* (*CRY1a* and *CRY1b*), one *CRY2* and one *CRY-DASH*. *Arabidopsis* plants overexpressing wheat *CRY1a* and *CRY2* were susceptible to ABA and osmotic stress, in addition to expected hyperphotomorphogenic phenotypes [16,17]. *Brassica napus* harbours a single *CRY1* member, involved in photomorphogenesis, hormone and stress signalling pathways regulation [18,19]. Algae instead contain a special type of CRYs whose functions are not just limited to photomorphogenesis [20]. Furthermore, recent reports reveal the presence of a novel type of blue light photoreceptor involved in light signalling like plant CRYs, as well as in DNA repair like bacterial DNA photolyases [21]. Algal CRYs probably represent a connecting link or intermediate stage in the evolution between DNA photolyases in bacteria and CRYs in higher plants.

During the day, all organisms are exposed to dial environmental rhythms: The daily transition from light to dark and the daily fluctuation of temperature. Specific light and temperature sensors enable the organisms to sense and respond to these changes, maintaining homeostatic balance [22]. In order to anticipate daily changes, several organisms have developed an internal timing mechanism known as the “circadian clock” that allows them to anticipate and align internal biological processes with these daily rhythms [23,24]. Circadian clocks consist of the input signals from the environment that reset the clock, the central oscillator that maintains a roughly 24-h rhythm independently from input signals, and the output signals that generate daily rhythms in physiology. Circadian clocks provide organisms the ability to synchronize their internal physiological responses with the external environment by a process termed “entrainment”. This process occurs through the perception of internal and external stimuli. In most living organisms, including plants, light stimuli perception is essential in entrainment and sustainment of circadian rhythms. In plants, the circadian clock controls an important array of processes including photosynthesis, thermomorphogenesis, hormone signalling, response to biotic and abiotic stress and flowering time [25]. Light signals are perceived and transduced to the central oscillator via at least four specialized classes of photoreceptors: CRYs detect blue light and UV-A, LOV-KELCH DOMAIN proteins also perceive blue light, PHYs primarily detect red light and far-red light, whereas UVR8 detects UV-B light [26]. Among these, CRYs play a fundamental and crucial role.

The focus of this review is to provide an overview on the role of plant CRYs in circadian clock entrainment, as well as highlighting the chromatin organisation of circadian clock genes and the reciprocal interaction between the clock and the cell cycle. Prior to that, the descriptions of plant CRY families and their molecular structures and physiological functions are reported.

## 2. The Cryptochrome Family in Plants

### 2.1. Cryptochrome Families

CRYs are one of the various photoreceptor classes identified in higher plants. They are activated by blue light and play a vital role in plant growth and development [27,28,29]. They are the first plant blue light receptors to be characterised at the molecular level [8,30]. CRYs were first discovered in the model plant *Arabidopsis* [8]. Soon after, CRYs were also found in other lineages, such as insects and mammals, acting as photoreceptors, transcriptional regulators or integral parts of the circadian oscillator [31,32,33,34].

Three different CRYs subfamilies, CRY1, CRY2 and CRY3 (or CRY-DASH), have been identified in *Arabidopsis* and other plants [8,35,36]. CRY1 was first identified in *Arabidopsis* to mediate blue light-dependent inhibition of hypocotyl growth [8]. *Arabidopsis* CRY1 locates and functions in both the nucleus and cytoplasm [37], whereas CRY2 seems to be an exclusively nuclear protein that completes its post-translational life cycle in the nucleus [38]. Instead, CRY3 mostly functions in chloroplasts and mitochondria organelles [36]. CRYs subfamilies and their composition, such as a number of paralogous genes, are species-specific. An exhaustive list of the CRYs subfamilies identified in model species was reported by Fantini and Facella [39]. Moreover, a comprehensive screening of available genomic resources and phylogenetic analysis, identified *CRY* paralogs in different plant species and highlighted that *CRY1* subfamily is present in all the phyla analysed, while *CRY2* only belongs to Magnoliophyta and *CRY3* is not present in Pteridophyta [39]. Recently, a thorough genome-wide search performed by Cao and co-authors using the *Arabidopsis* CRY amino acid sequence as query search against different genome data sets, identified *CRY* genes in different plant species [40]; a total of 94 *CRY1* and 68 *CRY2* candidate genes were retrieved from 58 and 50 plant genomes, respectively [40].

### 2.2. Cryptochromes Structure and Photochemical Activation

Structurally, all CRYs are globular flavoproteins with highly conserved sequences that share high structural homology to that of the light-dependent DNA photolyase, which is considered to be its evolutionary ancestor [41,42,43,44,45]. In fact, it has been speculated that DNA photolyases over the years of evolution have lost their DNA repair activity and instead have been modified to become light-activated signalling molecules.

CRYs consist of two core domains connected by a long inter-domain loop: The highly conserved amino-terminal photolyase homologous region (PHR) and the diversified carboxy-terminal extension (CCE, also referred to as CCT) of variable lengths and sequences. CCE domain is not present in photolyases and CRY3. The PHR domain, further subdivided into N-terminal α/β and C-terminal α subdomains or helical domain [5,46,47], binds two chromophores, cofactors that absorb light; one is the chromophore flavin adenine dinucleotide (FAD) and the other is the chromophore methenyl tetrahydrofolate (MTHF or pterin). They are absorbing mainly in blue/UVA range [48,49]. The FAD chromophore is non-covalently bound to the C-terminal α subdomain of the PHR within a hydrophobic pocket and plays a critical role in facilitating photoreduction [48,50]; MTHF which primarily functions in light harvesting, transfers its excitation energy to FAD, the catalytic cofactor. The C-terminal extension contains a DQXVP-acidic-STAES (DAS) domain conserved from moss to angiosperm. This domain is important for the nuclear/cytosol trafficking, protein-protein interactions and physiological functions of CRYs [41]. Earlier, the PHR domain was thought to act as the light-sensing domain and CCE domain as the effector domain. Most of the cryptochrome-interacting proteins, except CONSTITUTIVE PHOTOMORPHOGENIC 1 (COP1), physically interact with the PHR domain of CRYs, suggesting that both the PHR and CCE may act as effector domains [51]. The CCE domain interacts with COP1 ubiquitin ligase as part of light signalling pathway [52,53,54,55].

CRYs have conserved from photolyases their capacity to undergo light-dependent redox reactions of the flavin cofactor [43]. The mechanisms of CRY-mediated photoresponses in plants have been extensively studied over the last two decades [43,51]. Those studies suggest that plant CRYs undergo photoreduction and photo-oligomerization, triggering signal transduction to regulate gene expression and photoresponses. In darkness, plant CRY1 and CRY2 are present as inactive monomers containing oxidized FAD (FAD_ox_) in its inactive ground state. Under blue light conditions, the flavin undergoes photoreduction involving both electron and proton transfer to form neutral radical (FADH°) and reduced (FADH−) redox state intermediates. This photoreduction reaction occurs as a consequence of electron transfer to the excited state flavin via three evolutionarily conserved tryptophan residues called the “Trp-triad”. The resting state (FAD_ox_) is restored by a slow process of flavin reoxidation which occurs independently of light, requires molecular oxygen, and produces reactive oxygen species (ROS) [56]. The redox changes in FAD chromophore, during photochemical reactions, are accompanied by structural/conformational changes in the N-terminal/PHR domain and C-terminal/CCE domain of CRYs that are crucial for signalling.

Phosphorylation and dephosphorylation events are also common in the signal transduction pathway of CRYs. In fact, both CRY1 and CRY2 undergo specific blue light-induced phosphorylation of serine residues in the C-terminal domain [57,58]. The addition of negatively charged phosphoryl group leads to electrostatic repulsion of C-terminal domain from the negatively charged N-terminal domain. This leads to the “open”/active conformation of CRYs that favours the interactions with other proteins to bring about the blue light-mediated developmental changes [38,53,54,55,59].

Photoreduced CRY molecules undergo conformational changes in order to form physiologically active homo-oligomers (photobodies) that have a nuclear localisation. Photo-oligomerisation appears to increase the affinity of CRYs for other proteins, promoting their interaction with one or more of ~30 CRY-interacting proteins to form various cryptochrome complexes, collectively referred as cryptochrome complexomes [51]. The CRY-interacting proteins include the CRY-signalling proteins CRYPTOCHROME-INTERACTING BASIC-HELIX-LOOP-HELIX (CIBs) or PHYTOCHROME-INTERACTING FACTORS (PIFs), transcription regulators such as AUXIN/INDOLE-3-ACETIC ACID or CONSTITUTIVELY PHOTOMORPHOGENIC1/SUPPRESSORS OF PHYTOCHROME A (COP1/SPA) complex, the BLUE LIGHT INHIBITORS of CRYs (BICs) [60].

Recently, it was demonstrated that CRY1 and CRY2 undergo both light dependent homooligomerization (CRY1-CRY1 and CRY2-CRY2) and heterooligomerization (CRY1-CRY2) to carry out their molecular functions [61] Photo-oligomerisation is evolutionarily conserved in several plant and animal species [61]. The cryptochrome complexes ultimately alter the photoresponsive transcriptome or the stability of photoresponsive proteins to modulate plant growth and development. Key details of this model, including the structural differences that distinguish the physiologically active and inactive CRYs, remain unclear. In contrast to CRY1 and CRY2, it has been reported that CRY3 has no formation of homodimers so far [7,36].

Although CRYs were previously defined as photolyase-like proteins without DNA-repairing activity, this earlier definition is modifiable in light of the more recent findings that the CRY-DASH and algal CRYs have both DNA repairing and transcription-regulatory activities [62,63,64].

### 2.3. Cryptochromes Function

Plant CRYs are currently known to regulate a variety of blue light-induced responses [44], however it remains unclear exactly how these photoreceptors specifically regulate these complex photoresponses. Moreover, different plant CRYs have been found to exert overlapping biological functions [65,66]. CRY-mediated light responses in terrestrial plants include: Photoresponsive gene transcription [67], entrainment of the circadian clock [68,69,70], inhibition of the germination of dormant seeds [71], inhibition of hypocotyl elongation [8,72,73], stimulation of cotyledon expansion [66,74], anthocyanin accumulation [18,29], chloroplast development [75,76], modulation of gravitropic responses [77], root growth [78], guard cell development, stomata opening [79,80,81], regulation of shade avoidance [82], control of programmed cell death [83], pathogenic responses [84,85], promotion of floral initiation [35], regulation of fruit development and metabolic composition [13,86], leaf senescence [87], phototropism [88] and magnetoreception [89,90] regulation and likely additional photoresponses yet to be discovered.

*A. thaliana* has been used to study plant CRYs most extensively. Genetic analyses of *Arabidopsis cry1* and *cry2* mutants reveal that both CRYs are able to regulate hypocotyl elongation. CRY1 is primarily involved in anthocyanin accumulation and cotyledon expansion, while CRY2 plays a role in the circadian clock system and photoperiod-dependent flowering [5].

CRYs induce plant responses to blue light primarily through the transcriptional regulation of a great number of genes [43]. In *Arabidopsis*, CRY1 and CRY2 regulate the transcription of 5–25% of genes, during seedling development under blue light conditions [77,91,92,93]. Further studies highlighted the ability of CRYs in regulating the expression of numerous genes in other plants such as tomato [94,95] and *Brassica napus* [19].

Two mechanisms, both involved with blue light-dependent protein-protein interactions of CRYs and the signalling proteins, have been elucidated for CRY-mediated transcriptional regulation: light-dependent modulation of transcription (e.g., the CRY-CIB complex) and light-dependent suppression of proteolysis (the CRY-COP1-SPA complex). The first mechanism concerns direct binding of CRY2 with CIB1 proteins to regulate their function, promoting photoperiodic flowering [96]. It is the first blue light-specific cryptochrome-interacting protein identified in plants [96]. CIB1 positively regulates floral initiation through CRY2, interacting with the promoter of the FLOWERING LOCUS T (FT) gene. *FT* encodes a mobile transcriptional regulator that migrates from leaves to apical meristem to activate transcription of floral meristem identity genes [97]. However, recently it has been reported that CIB proteins directly interact with CONSTANS (CO) to promote floral initiation [98], implying that CRY2 might affect CIB–CO interaction. Moreover, the interaction between CRY2 and CIB1 also regulates other physiological processes as leaf senescence [87].

Plant CRYs interact with a group of bHLH transcription factors, known as PIF 1 to 7 proteins, which are phytochrome interacting and G-box-binding transcription factors related to the CIB proteins [99,100]. Although PIFs proteins are best known for their roles in the phytochrome-mediated responses, they appear to act as systems integrators that combine different signals, including hormones, sugar, circadian timing, and temperature [100,101]. Two recent studies demonstrate that photoexcited CRYs interact with PIF4 and PIF5, via the N-terminal PHR domain of CRYs and the N-terminal domain of PIFs in the region distinct from the phytochrome-binding motif. The CRY-PIF interaction inhibits the activity of PIF4 and PIF5, resulting in regulation of hypocotyl elongation under low blue light conditions [82]. Blue light and temperature are two abiotic factors acting as two key environmental signals that profoundly affect plant growth and development responses. In *Arabidopsis* CRY1 represses high temperature-induced hypocotyl elongation through PIF4. CRY1 physically interacts with PIF4 in a blue light-dependent manner to repress the transcription activity of PIF4. Because PIF4 also plays a role in ambient temperature responses, PIF4 appears to be the molecular basis of cross-talk among blue and red light and ambient temperature signal pathways [102]. In addition to PHYs, CRYs have been proposed to mediate shade avoidance responses [103]. In fact, it was proposed that, under low blue light, the decreased activity of CRYs weakens their interaction with PIFs, allowing the PIF proteins to promote stem elongation that presumably helps plants grow out of the unfavourable shade condition under canopy [103]. 

The second mechanism involved in CRY-mediated transcriptional regulation regards the action of the COP1-SPA complex. It is a central regulator of plant photomorphogenesis and is best known for its function as substrate for the cullin 4-based E3 ubiquitin ligase, CUL4^COP1/SPAs^, involved in ubiquitination and degradation of different light-signalling proteins [104]. It is well known that CRYs mediate blue light suppression of the E3 ubiquitin ligase COP1 and COP1-dependent proteolysis to affect gene expression [92,105]. Structural analysis has recently shown that the VP/DAS motif of the CCE domain of *Arabidopsis* CRY2 directly interacts with COP1 to exert CRY functions [106]. CRY1 mediates blue light suppression of the COP1-dependent degradation of the bZIP transcription factors LONG HYPOCOTYL5 (HY5), which in turn regulate transcription of genes required for the de-etiolation response [105]. HY5 binds to the promoters of several light-regulated genes and, in this way, it promotes photomorphogenic development [107]. Conversely in the dark, the COP1/SPA complex targets HY5 for ubiquitination, thereby inducing proteasomal degradation. Light inactivates the COP1/SPA complex allowing the accumulation of HY5 in the nucleus and triggering photomorphogenic responses [108]. Similarly, the blue light-dependent binding of CRY2 to SPA1 also inactivates COP1/SPA1 complexes [109], causing CO accumulation. CO binds to the promoter of the florigen coding gene *FT*, inducing the expression of *FT* under long day conditions and regulating photoperiodic flowering [110,111].

## 3. Cryptochromes and Circadian Clock

**Figure 1:**
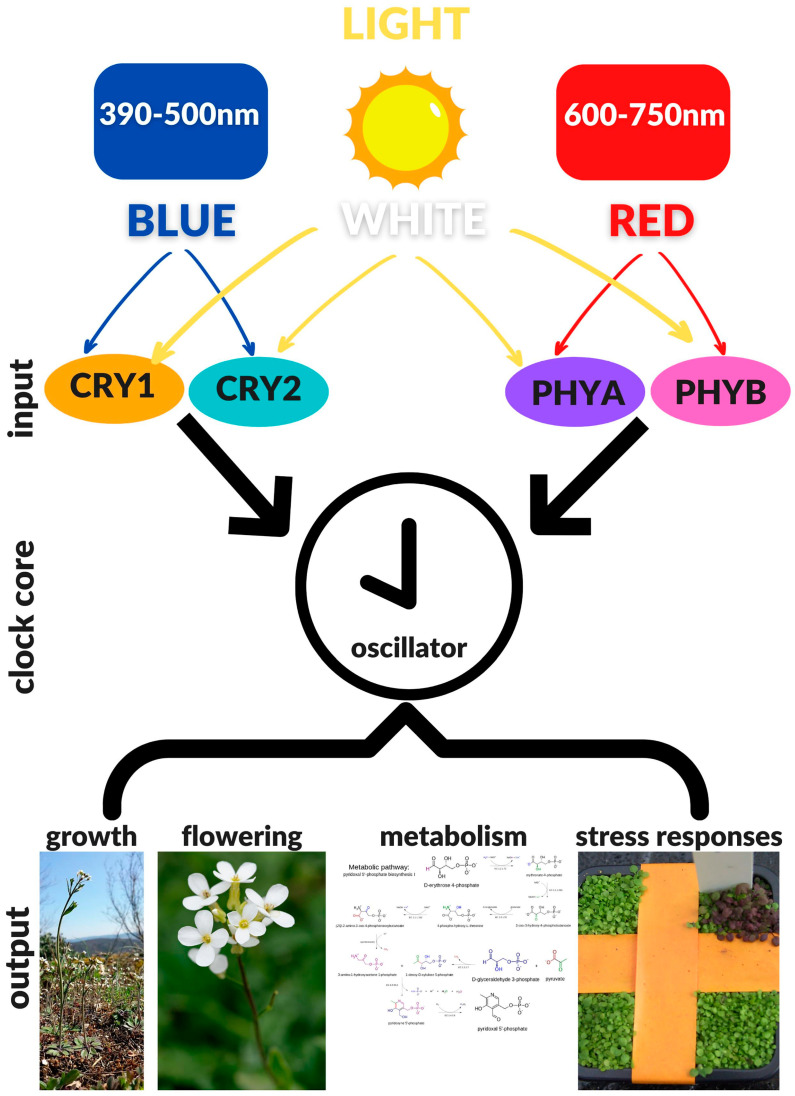
Schematic representation of the *Arabidopsis* circadian clock, depicting the three principal components of the circadian machinery: The INPUT system—cryptochromes (CRYs) and phytochromes (PHYs)—that receives light stimuli and entrains the clock; the CLOCK CORE that generates 24-h self-sustained oscillations, also in absence of environmental stimuli; the OUTPUT system that adapts the developmental and physiological responses of the plant to the circadian fluctuations. Images from: https://commons.wikimedia.org/wiki/File:Arabidopsis_thaliana_sl10.jpg; https://commons.wikimedia.org/wiki/File:Metabolic_pathway-_pyridoxal_5%27-phosphate_biosynthesis_I_v_2.0.svg; https://commons.wikimedia.org/wiki/File:Arabidopsis_thaliana_JdP_2013-04-28.jpg; https://commons.wikimedia.org/wiki/File:Plants_that_change_color_and_mark_buried_explosives.jpg (accessed on 18 March 2021).

The Earth has rotated about its axis of inclination since its formation—a rotation generating the daily transition between light and dark with a period of approximately 24 h. The alternation between day and night results in dramatic variations in certain environmental conditions, including light quality and quantity, and temperature oscillations. These daily fluctuations of fundamental environmental factors heavily influence life and as a way of sensing and, consequently, responding to these changes, organisms have evolved various light and temperature sensors. It is evolutionarily advantageous for organisms to anticipate daily environmental changes and prepare for them accordingly. To synchronize their internal physiological processes with the predicted environmental variations, most organisms, including plants, have evolved an internal molecular time-keeping mechanism called the “circadian clock” [23,24]. The circadian clock is able to perform self-sustained oscillations with a period close to 24 h even under constant environmental conditions. Recent studies have demonstrated that the circadian clock runs cell-autonomously, with each cell capable of maintaining its own rhythmicity of about 24 h [112]; in this way, multicellular organisms are able to keep tissue and organ-specific clocks [113]. The most important elements of circadian machinery are: The environmental input signals that act to reset the clock; the central oscillator that maintains an approximately 24-h rhythm, even in the absence of input signals; the output signals that generate daily rhythms in development and physiology (Figure 1). The phase and the amplitude of the central oscillator are modulated by the intensity and quality of light stimuli [68,69].

### 3.1. Central Oscillator

In plants, the clock core that generates the 24-h rhythm includes a highly complex network of genes that act as repressors and/or activators, creating multiple interlocked transcriptional feedback loops formed by transcription factors [114,115]. The clock genes present temporal waves of expression, with peak activity at different times of the day. Moreover, they regulate each other’s transcription, modulating several fundamental physiological processes such as flowering time, phytohormone synthesis, growth control, metabolic activities, biotic and abiotic stress responses, etc. MYB-like transcription factors CIRCADIAN CLOCK ASSOCIATED 1 (CCA1) and LATE ELONGATED HYPOCOTYL (LHY) show a peak of expression near dawn; they repress the transcription of *PSEUDORESPONSE REGULATOR* (*PRR*) (9,7,5 and 1, also known as *Timing of CAB expression 1* (*TOC1*)) genes that are expressed in the afternoon, with *TOC1* displaying maximum expression at dusk [116,117,118]. In turn, the PRRs repress *CCA1/LHY* expression, creating a feedback loop [116,119]. CCA1 and LHY are able to repress the transcription of many clock genes, binding to a cis-motif, the “evening element” (EE), present in the promoter of several genes with a peak of expression at the evening. In addition to *PRR* genes, CCA1/LHY function of transcriptional repressors also concern some genes of the so-called “evening complex” as *ELF3* (*EARLY FLOWERING3*), *ELF4* (*EARLY FLOWERING 4*), and *LUX* (*LUX ARRHYTHMO*). These three genes show a maximum of transcription at night; at this time of day they repress several morning and afternoon genes, giving rise to a further feedback loop [115]. A second group of MYB-like transcription factors made by the REVEILLE family (4, 6 and 8) promotes the transcription of evening complex and *PRRs* genes, in particular, *TOC1* [120,121,122]. It was recently shown that RVE8 binds to LNK1 and LNK2 proteins (NIGHT LIGHT-INDUCIBLE AND CLOCK-REGULATED1 and 2), forming a complex capable of binding to *PRR5* and *TOC1* promoters and activating their transcription in the afternoon [123]. In turn, PRRs, activated by REV8, repress the transcription of *CCA1/LHY* until dawn. During the night, TOC1 promotes the degradation of the F-box protein ZTL [124]. GIGANTEA (GI) stabilizes ZTL protein during the day, inhibiting its interaction with TOC1 until night [125].

### 3.2. Cryptochromes in the “Input” System

Light variations over a day–night cycle are robust and hence drive the entrainment of the clock. For example, the transition from dark to light occurring at dawn is used by plants as a time setting checkpoint [126].

In *Arabidopsis* both CRY1 and CRY2 play an important role in driving light input to the circadian clock [69]. In plants, the circadian clock can be studied through several phenotypic responses as leaf movements, Ca^2+^ oscillations or luciferase report fluorescence (LUC) [127,128,129]. The overexpression of CRY1 caused a shorter period of *chlorophyll A/B binding 2::LUCIFERASE* (*CAB2::LUC)* expression rhythms with respect to wild-type plants, under low blue light intensities [68,89]. The *cry* mutants are altered in the circadian clock [68]. Under blue light in both high and low fluence rates, the *cry1* mutant presents a longer period of the circadian rhythm, while the *cry2* mutant shows a slight shortened period but only under low fluence conditions. Nevertheless, the double mutant *cry1*/*cry2* exhibits a longer period that is not dependent by the fluence rate, indicating that CRY1 and CRY2 may act in an additive and redundant modality in regulating light input to the clock. It is plausible to hypothesize that the effect of the lacking CRY2 appears evident only in the absence of CRY1. Moreover, the long-period phenotype of *cry1*/*cry2* was also described in Ca^2+^ oscillations and leaf movements [128,130]. Besides, the blue light induced phase shift of leaf movement rhythm is strongly reduced in *cryl* and *cry1/cry2* mutants, showing that CRY1 and CRY2 regulate blue light entrainment of the *Arabidopsis* circadian clock. However, the double mutant *cry1*/*cry2* is still rhythmic, demonstrating that CRYs are not part of the oscillatory core of the clock, as shown in mouse [34,69,131,132], although *CRY1* and *CRY2* transcript levels oscillate with a circadian rhythm under a constant light condition [133]. It is well known that in white light that includes multiple light wavelengths, CRYs interact with PHYs in order to transmit environmental information to the clock [69]. In wild-type *Arabidopsis*, *CAB2* period declines if the light intensity rises; it was demonstrated that *cry2* mutants are deficient in a white light response as they have a *CAB2* promoter period increase when they are exposed to light with high fluence rate [134]. The *cry2* mutant period increase was not found in red or blue light, showing that CRY2 needs multiple wavelengths of light simultaneously and PHYB to be active [134]. As mentioned above, CRY2 is functional active under multiple light wavelengths (as is the case under white light), indicating a role of this CRY for the activation of PHYs [134]. Indirect evidence for the interaction between CRYs and PHYs in regulating circadian clock input is provided by the synchronisation of their expression pattern [135]. Moreover, it was demonstrated that CRY2 needs the active Pfr form of PHYs for its expression and, in turn, CRY2 inhibits PHYB expression. PHYA is able to phosphorylate both CRY1 and CRY2, through its kinase activity. Thus, a plausible hypothesis is that PHYs and CRYs cooperate in response to white light, even if the exact molecular and functional interconnections between these two families of photoreceptors are not yet completely clear. In addition to that, other photoreceptors could have an important impact in resetting the clock, as suggested by the fact that the quadruple mutants *phya/phyb/cry1/cry2* are still rhythmic under constant light conditions [132].

In the past, many studies suggested a further role for CRYs in mediating green light physiological responses. It was demonstrated that the overexpression of CRY1 gives rise to a detectable increase of plant sensibility to green light [9]. Moreover, the inhibition of hypocotyl elongation under blue light is reversed by green light. This phenomenon is probably caused by the inversion of the blue light-mediated CRY1 degradation, that is inhibited by green light [136]. Recently, studies demonstrated a role of CRYs in resetting the clock also under green light stimulation [137]. The authors show that low fluence rates of green light are able to entrain and maintain circadian oscillations in *Arabidopsis* through the action of both CRYs. However, the role of CRYs in modulating green light-mediated clock reset is still unclear and further studies are needed to unravel this complex aspect.

The integration between the input system, regulated by photoreceptors, and the clock core, formed from several complex gene and protein interconnections, gives rise to a number of physiological plant responses to environmental stimuli: hypocotyl growth, flowering time, stress responses, phytohormone synthesis, etc. Understanding the multifaceted crosstalk between the molecular actors of the circadian clock system appears to be of extreme importance for improving our knowledge of plant life and, consequently, improving the quantity and quality of different crop species. In this context, the action of CRYs still remains nebulous and many aspects of their role in resetting circadian clock demand clarification. Further studies on this aspect of CRYs function in plant will shed light not only on this family of photoreceptors, but also on the main physiological aspects of circadian regulation in the plant life cycle.

## 4. Cell Cycle, Epigenetics and Circadian Clock

The cell cycle is a series of events divided in four phases: G1 (Gap 1 also called the post mitotic growth phase), S (duplication of DNA and some organelles), G2 (Gap 2 also called pre mitotic growth phase that include protein synthesis and rapid cell growth) and M (Mitotic transition), and the subsequently partitioning of the cytoplasm and other components into two daughter cells in a process called cell division.

Two key classes of regulatory molecules, cyclins and cyclin-dependent kinases (CDKs) determine a cell’s progress through the cycle. The circadian clock and cell cycle as separate pathways have been well documented in plants. Elucidating whether these two oscillators are connected is critical for understanding plant growth and development. Despite its biological relevance in plants, the possible connection between the circadian clock and the cell cycle remained elusive until a recent study by Fung-Uceda and co-authors [138]. They show that in order to accurately regulate the number and size of plant cells in synchrony with the environment, the circadian clock sets the time of the cell cycle. In plant cells the clock is able to control not only plant growth but also tumour progression through DNA replication. The activation of CDKs is responsible for the cell proliferation through progression of the mitotic cycle which associate with specific cyclins (CYCs) to control the G1 to S and the G2 to M transition phases [139]. To ensure proper control of the cell cycle there are critical checkpoints at the transitions. D-type CYCs (CYCD) and A-type CDKs (CDKA) survey the G1-S-phase transition [140] and contribute to M-phase entry [141]. To allow cells to progress into S-phase, the key regulatory event for cell-cycle progression is licensing DNA for replication.

The progressive formation of pre-replicative complexes is constituted by a number of proteins including the Origin Recognition Complex (ORC), CELL DIVISION CONTROL 6 (CDC6), ARABIDOPSIS HOMOLOG OF YEAST CDT1 (CDT1a) and minichromosome maintenance (MCM). In *Arabidopsis*, *CDC6* is upregulated at the G1-S transition, reaching a peak early in S-phase [142]. The S-phase relies on a balance between the inhibition of the E2F/DP transcriptional activity by the hypo-phosphorylated retinoblastoma-related protein (RBR) and RBR phosphorylation by the CDKA-CYCD kinase activity, which relieves their repression [141]. E2Fa/b activates the expression of genes involved in DNA synthesis and replication including *CDC6* and *CDT1* [143]. Their transcriptional and post-transcriptional regulations are the key to sustaining the balance between cell proliferation and differentiation [139].

**Figure 2:**
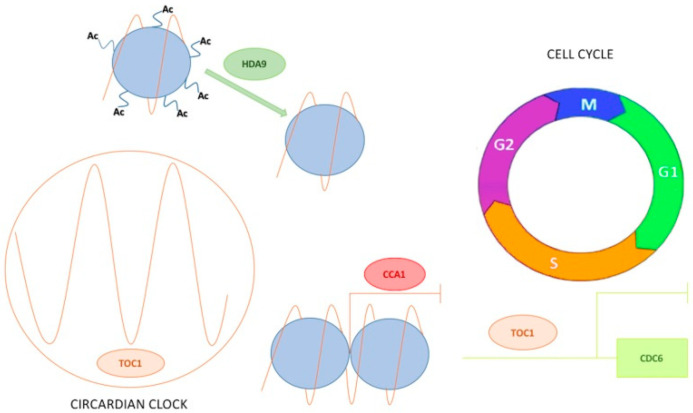
Epigenetic regulation of clock components and their role during cell cycle. (**Left**) Histone modifications modulate the rhythmic expression of clock genes: at dusk TIMING OF CAB EXPRESSION 1 (TOC1) expression increases due to hyperacetylation. Once TOC1 reaches its peak, the downregulation is accompanied by the action of the histone deacetylase HDA9 which facilitates CIRCADIAN CLOCK ASSOCIATED 1 (CCA1) mediated repression at the end of the day. (**Right**) At the transition between Gap 1 (G1) and duplication of DNA (S) phase, TOC1 regulates the progression of the cell cycle by repressing the expression of *CELL DIVISION CONTROL 6* (*CDC6*) responsible for DNA replication.

In *Arabidopsis,* recent studies demonstrated that the circadian clock through TOC1 regulates the cell cycle speed [138]. The circadian clock modulates cell division, exerting the DNA pre-replicative machinery regulation and thus controls not only normal growth but also tumour development primarily during the S-phase. TOC1 regulates the proper timing of the G1- to S-phase transition, as indicated by the relative duration of the G1 and S-phases as well as by the delayed S-phase entrance to the promoter of the DNA replication factor *CDC6* to repress its diurnal expression (Figure 2). Cell size and number, somatic ploidy, organ size and the overall plant growth are coordinated by the clock in synchronisation with the environment. The function of TOC1 in the mitotic cycle resembles that of the mammalian circadian component NONO, an interacting partner of the clock protein PERIOD that circadian-gates the S-phase in fibroblasts [144]. It would be interesting to understand whether in addition to TOC1, other clock components, such as CRYs, can contribute to the regulation of the cell cycle at different cell cycle phases in plants. Notwithstanding the fact that both circadian clock and cell cycle are strongly conserved among all eukaryotic kingdoms, so far only very few studies have investigated the relation between them.

The chromatin status of the circadian clock components represents an important level of regulation that underlies the rhythm of the transcriptional changes occurring during plant development [145,146,147]. Chromatin is usually organised via different levels of regulation, including modifications of the DNA sequence, histone tails and histone variants, and finally by the presence/absence of short interfering RNA molecules [148]. In particular, histone tails can be chemically altered by adding and removing acetyl CoA and methyl groups. The modulation and the position of such modifications can be correlated with an enhancement or reduction of gene expression [149,150].

Over the years, it has been reported on how changes in the epigenetic state of clock genes modulate their expression. The first remark was observed for *TOC1* whose expression was correlated with the H3 acetylation levels at its promoter region [151]. In particular, *TOC1* expression during the day was correlated with an increase in histone acetylation. Conversely at dawn, *TOC1* repression by CCA1 was facilitated by H3 hypo-acetylation [151] (Figure 2). CCA1 repressive function is counteracted by the MYB transcription factor RVE8 during the day, which then leads also to an increase in *TOC1* acetylation marks [123]. Recently, the Histone Deacetylase 9 (HDA9) and the Evening Complex member ELF3 were found to form a complex that specifically binds *TOC1* promoter and regulates its expression during the declining phase [152]. More specifically, the presence of ELF3 facilitates HDA9 binding on *TOC1* promoter thereby inducing histone deacetylation [153] (Figure 2). Together with H3 acetylation, other histone marks are associated with *TOC1* promoter. The positive mark H3K4me3 was also found to accumulate during the day and to prevent any unnecessary CCA1 binding and ensuring the required *TOC1* rhythmic expression. Accumulation of H3 methylation marks depends mostly on the histone methyltransferase SET DOMAIN PROTEIN 2 (SDG2/ATXR3) as *TOC1* expression was largely affected in case of misexpression of the enzyme [154].

A similar type of regulation was also found for other clock components, including *CCA1*, *LHY*, *PRR9*, *PRR7* and *LUX*. The HISTONE ACETYLTRANSFERASE OF THE TAFII250 FAMILY 2 (HAF2) contributes to the circadian oscillation primarily at midday when it reaches its peak of expression and promotes H3 acetylation at *PRR5* and *LUX* loci, enhancing their expression [155]. Conversely, SAP30 FUNCTION-RELATED 1 (AFR1) and AFR2 which are part of the SIN3-HDAC complex can directly deacetylate *CCA1* and *PRR9* promoter regions modulating their expression at dusk. Moreover, *CCA1* and *PRR9* were found to be upregulated in *afr1*/*afr2* double mutant [153]. Histone H2B ubiquitination (H2Bub) is another modification that has been associated with H3 methylation and with the increase of RNA polymerase II activity [156]. Interestingly, gene expression studies using *hub1-1* mutant revealed a reduction in the amplitudes of the expression peaks of several clock genes. Furthermore, chromatin immunoprecipitation of specific targets showed a strong correlation for the deposition H2B and H3K4me3 marks along *CCA1*, *Light-Harvesting Complex* (*LHC*) (*LHCB2.1*), *Ribulose Bisphosphate Carboxylase Small chain* (*RbcS*), *ELF4* and *TOC1* loci [157].

In plants, histone ubiquitination genetically interacts with the “FACILITATES CHROMATIN TRANSCRIPTION” (FACT) complex, indicating a putative role of transcriptional elongation on the circadian clock [158]. Together with active histone marks, also repressive histone modifications are correlated with clock components. For instance, H3K36me2 appeared to negatively correlate with the expression of clock genes [159]. The modulation of central clock components is characterised by interlocked feedback loops that are displayed by multilayer repressive states. Wang and collaborators were able to show that several members of the PRRs family can interact with the GROUCHO (GRO)/dTMP-UPtake (TUP1) corepressor complex that also includes TOPLESS and histone deacetylase 6 or 19 [160]. This complex is located on the promoter regions of *CCA1* and *LHY* thereby repressing their expression. Such repression is alleviated by treatments with the histone deacetylation inhibitor TRICHOSTATIN A, suggesting that HDA6 or 19 are required for function. Co-immunoprecipitation studies demonstrated that TOPLESS works as an adaptor between PRR9 and HDA6, further demonstrating the presence of a PRR-TPL-HDA repressive complex at the promoters of the *CCA1* and *LHY* genes [160]. Together with HDA6, yeast two hybrid screening has shown that CCA1 can also interact with histone deacetylase SIRTUIN1 (SIR1) [161]. Moreover, the analysis of the circadian expression of over 100 histone modifiers has shown that more than 10% of them display a rhythmic oscillation. Among those histone deacetylases, demethylases and methyltransferases were included [161]. However how these genes function within the plant clock, still remains to be determined.

Consistent with clock features, in turn, chromatin regulators are also modulated by circadian rhythms. For instance, CCA1 can directly bind the promoter of *PICKLE*, which encodes for an ATP-dependent chromatin remodeller, which plays a major role in hypocotyl elongation under high temperatures [162]. In this case CCA1 works as a positive regulator of expression as *PICKLE* appears to be downregulated in *cca1* mutant. In a similar manner, CCA1 and LHY bind *JUMONJI30* (*JMJ30*) DNA regions to repress its transcription [163]. The JUMONJI C (JMJC) domain containing proteins are considered histone demethylases [164]. By functional analysis JMJ30 was revealed as a new clock component since its expression oscillates with a circadian rhythm and analysis of the *CAB2::LUC* reporter gene showed a shorter rhythm in *jmj30* mutant compared to wild-type [163].

Parental effect that regulates heterosis has been found to have a circadian regulation primarily through DNA methylation. In particular, in allotetraploids generated between *A.* *thaliana* and *A.* *arenosa*, the *CCA1* allele, which was maternally transmitted, was more repressed because of CHH DNA methylation sequence context in the promoter site [165]. However, it is not yet clear which DNA methylation pathway is responsible for *CCA1* expression.

### Perspectives on the Link between Cryptochromes and Chromatin: A Newly Discovered Function?

As mentioned above, CRYs are plant blue light photoreceptors that modulate the progression and the maintenance of the circadian rhythms [137]. Although not much is described on the relationship between CRYs and chromatin in plants and how they may interact with chromatin remodellers, in the mammalian field there are several reports suggesting that the epigenetic regulation of CRYs is important for its role as clock component. For example, in patients with Chronic Lymphatic Leukaemia (CLL), *CRY1* appears to be downregulated in a clock dependent manner [166]. A follow up study describing DNA methylation analysis of blood samples from over hundred CLL patients showed that *CRY1* low expression was linked to an aberrant CpG pattern of *CRY1* promoter [167]. In another study using mammalian cell cultures, it was proved that CRY1 can associate with DNA regions of different receptors to initiate their expression, therefore deviating from its role as circadian clock repressor and indicating its ability to bind chromatin [168]. In plants, studies have shown that CRY2, in particular, can modulate chromatin compaction [169]. In details, plants grown under low white light showed a decrease in heterochromatin index (HX) which is usually monitored to determine changes in chromatin compaction. In particular, analysis of different photoreceptor mutants indicated a significant higher HX after low light treatment specifically for CRYs mutants. In addition, the levels of CRY2 protein were enhanced in low light conditions, suggesting that CRY2 abundance determines the level of compaction [169]. Recent work has also indicated that CRY2 can physically associate with genomic regions to regulate their expression. More specifically, ChIP-seq experiments showed that under low blue light conditions (LBL), CRY2 genomic DNA targets overlap with those bound by PIF 4 and 5 with a significant enrichment for the E-box motif. Among the genes with a higher expression in LBL, the authors describe *ARABIDOPSIS THALIANA HOMEOBOX PROTEIN 2* (*ATHB2*) and *XYLOGLUCAN ENDOTRANSGLUCOSYLASE/HYDROLASE 33 (XTH33)*. These findings suggest that *PIF4/5* and *CRY2* are present at similar genomic loci to initiate gene expression. Consistent with these results, the overexpression of ATHB2 and XTH33 caused an increase in hypocotyl growth compared to the wild-type under LBL [82].

Another contribution demonstrated that CRY1 can physically interact with members of the SWI2/SNF2-Related 1 (SWR1) chromatin remodelling complex responsible for the deposition of the histone variant H2A.Z [170]. In particular, the authors showed that under blue light conditions CRY1 interacts with SWR1 complex subunit 6 (SWC6) and Actin-related protein 6 (ARP6) thus promoting H2A.Z deposition on HY5 targets and inhibiting hypocotyl elongation in Arabidopsis.

Despite the evidence that CRYs can indeed act as transcriptional regulators and associate with chromatin, such a role in the plant circadian clock still remains unexplored. Further work is therefore required to determine whether CRYs or CRYs containing complexes can associate with genomic regions of clock component genes.

## 5. Conclusions

CRYs play several fundamental roles in plant development and physiology. Thanks to many studies arising in the last few years, their complex interconnections with different plant molecular networks have been elucidated. Cryptochrome resetting of the circadian clock is certainly one of the most interesting aspects of the activity of this family of photoreceptors in plants, with their role in epigenetic and cell cycle regulation still to be determined. For this reason, our knowledge of their action in plant species other than *Arabidopsis* is still scarce. In particular, the role of the circadian clock in the plant cell cycle remains to be fully explored. Altogether, understanding cryptochrome influence on the circadian clock, epigenetic mechanisms and cell cycle could be extremely important for crop species with relevant economic value. Based on these premises, future research should focus on extending circadian molecular and genetic studies to high added-value plant species.

## Data Availability

Not applicable.
